# Same margin, different lesion: margin subtype, glandular involvement, and endocervical curettage status as predictors of residual CIN2+ at repeat loop electrosurgical excision procedure: An 11-year retrospective cohort study

**DOI:** 10.1590/1806-9282.20261101

**Published:** 2026-11-09

**Authors:** Yunus Emre Purut, İlayda Gercik, Anıl Doğukan Tutal, Sercan Kantarcı, Ceren Sağlam Purut, Göktürk Han Doğan

**Affiliations:** 1 S.B.U. İzmir Tepecik Training and Research Hospital, Department of Obstetrics and Gynecology – İzmir, Türkiye.; 2 İzmir City Hospital, Department of Perinatology – İzmir, Türkiye.; 3 İzmir Atatürk Training and Research Hospital, Department of Perinatology – İzmir, Türkiye.

**Keywords:** Cervical intraepithelial neoplasia, Neoplasm, Surgical margin, Residual disease, Human papillomavirus, Re-excision

## Abstract

**OBJECTIVE:**

The aim of this study was to assess how initial loop electrosurgical excision procedure margin subtype, glandular involvement, endocervical curettage findings, and specimen size relate to re-loop electrosurgical excision procedure pathology and receipt of further surgery.

**METHODS:**

In this retrospective cohort study, 287 patients undergoing re-loop electrosurgical excision procedure at a tertiary center (2015–2025) were reviewed. Initial margin status was classified as margin continuity, margin adjacency, or non-evaluable margin. Associations with re-loop electrosurgical excision procedure pathology, residual CIN2+ disease, and receipt of a third surgical intervention were assessed using chi-square/Fisher-Freeman-Halton tests, Cohen's kappa, and multivariate logistic regression.

**RESULTS:**

Initial and re-loop electrosurgical excision procedure pathology showed a significant group-level association (p=0.039) but no case-by-case agreement beyond chance (kappa=-0.009, p=0.754); most patients with initial CIN2/3 had CIN1 at re-loop electrosurgical excision procedure. Margin adjacency independently predicted residual CIN2+ disease at repeat loop electrosurgical excision procedure (OR 11.045, p=0.049), as did glandular involvement (OR 1.938, p=0.035). Among patients with negative re-loop electrosurgical excision procedure margins, residual CIN2+ disease was identified within the specimen in 27.1%, and human papillomavirus test negativity at the first follow-up visit was 88.2%, varying by genotype. Positive margin (OR 333.33, p<0.001) and positive endocervical curettage (OR 41.67, p<0.001) at repeat loop electrosurgical excision procedure were the strongest predictors of receiving a third surgical intervention.

**CONCLUSION:**

Margin adjacency and glandular involvement independently predicted residual CIN2+ at repeat loop electrosurgical excision procedure, whereas positive margins and positive endocervical curettage at re-loop electrosurgical excision procedure were strongly associated with receipt of a third surgical intervention. Post-loop electrosurgical excision procedure follow-up should integrate margin subtype, glandular involvement, endocervical curettage findings, and human papillomavirus surveillance rather than rely on margin status alone; genotype-specific differences in human papillomavirus test negativity require confirmation in larger cohorts.

## INTRODUCTION

Cervical cancer remains one of the most common gynecological malignancies worldwide, and nearly every case traces back to persistent high-risk human papillomavirus (HPV) infection, with genotypes 16 and 18 predominating^1,2^. Cervical intraepithelial neoplasia (CIN) is the histological precursor spectrum, ranging from low-grade lesions prone to spontaneous regression to high-grade lesions (CIN2/3) that carry a meaningful risk of progression to invasive carcinoma if untreated^3,4^. Although active surveillance is increasingly used for selected young women with CIN2, CIN3 and persistent or progressive CIN2 still warrant excisional treatment^5^.

Loop electrosurgical excision procedure (LEEP) is the most widely used excisional technique for high-grade CIN, given its favorable safety profile, low cost, and ability to provide a specimen for margin assessment^6^. Despite this, a substantial proportion of patients have disease at or near the excision margin, and margin status remains one of the strongest predictors of treatment failure, with meta-analytic data showing markedly higher recurrence risk with positive versus negative margins^7^. This creates a clinical dilemma. Repeat excision or hysterectomy is often considered for positive margins, yet many such patients do not, in fact, harbor residual disease, raising concerns about overtreatment given the known association between excisional treatment and adverse obstetric outcomes such as preterm birth^8^.

Beyond binary margin status, features such as the extent of margin involvement, glandular involvement, ECC findings, and specimen size have been proposed as additional prognostic markers^9,10^. However, most literature still relies on binary margin classification, and little is known about how more granular categories, margin adjacency without transection, margin continuity, and non-evaluable margins due to thermal artifact, relate to outcomes at repeat excision^7,11^. Data are also limited on patients requiring a third surgical intervention and on genotype-specific HPV clearance after negative re-LEEP margins.

We therefore conducted a retrospective cohort study of patients undergoing re-LEEP at a tertiary referral center over an 11-year period, evaluating whether findings at repeat excision can be anticipated from the initial specimen at all, and how detailed initial margin subtypes, glandular involvement, ECC findings, and specimen characteristics relate to re-LEEP pathology and receipt of further surgery, to identify which initial histopathological features most reliably predict clinically meaningful residual disease.

## METHODS

### Study design and setting

This retrospective, non-interventional cohort study was conducted at S.B.U. Izmir Tepecik Training and Research Hospital, University of Health Sciences, Izmir, Turkiye, in accordance with the Declaration of Helsinki, and was approved by the institution's Non-Interventional Research Ethics Committee (approval no. 2025/12-07, January 5, 2026). Given the retrospective design, individual informed consent was waived.

### Study population

We reviewed patients who underwent colposcopy-directed biopsy and subsequent LEEP for histologically confirmed CIN at our center between January 2015 and December 2025, and who underwent a second (re-)LEEP because of a positive or margin-adjacent surgical margin, a positive ECC, an indeterminate margin, or high-grade cytology on follow-up (positive margin in 202 patients, combined positive margin and ECC in 47, indeterminate margin in 31, isolated positive ECC in 5, and atypical squamous cells, cannot exclude a high-grade squamous intraepithelial lesion (ASC-H) cytology in 2). At histopathological classification, these indications corresponded to margin continuity or margin adjacency in 251 patients and to a non-evaluable margin in 36. Patients with a clear negative initial margin, those who declined re-LEEP, those with invasive carcinoma at initial LEEP or biopsy, those who underwent primary hysterectomy without a second excisional procedure, and those with incomplete records were excluded. A minimum interval of 6 weeks was required between the initial LEEP and re-LEEP; after this minimum interval, re-LEEP was scheduled for the earliest possible time after this period.

### Data collection and variable definitions

Demographic characteristics, baseline HPV genotype and cytology, colposcopy-directed biopsy results, and initial LEEP findings (histopathology, glandular involvement, ECC, margin status, specimen size) were extracted from institutional records. Margin status at initial LEEP was classified as margin continuity (CIN extending continuously to the cut edge), margin adjacency (CIN within 1 mm of the margin without unequivocal transection), or non-evaluable margin (status unassessable, typically due to thermal artifact)^12,13^. Margin status was assigned from the routine histopathological report generated at the original procedure rather than by re-review of specimens for this study; when thermal artifact was present but the reporting pathologist was nonetheless able to render a margin call, that call determined classification as continuity or adjacency, and only specimens for which no margin call could be made were classified as non-evaluable. Margin distance was assessed visually by the reporting pathologist under routine light microscopy; a standardized micrometer-based measurement protocol was not used, and inter-observer variability in this assessment was not formally evaluated. Findings at re-LEEP included histopathology, glandular involvement, ECC, and margin status; patients were further stratified by whether a third intervention (repeat LEEP, cold-knife conization, or hysterectomy) was performed. ECC was routinely performed after the excisional procedure. Positive ECC was defined as CIN2 or worse on endocervical curettage. ECC performed immediately after LEEP carries a theoretical risk of contamination by excision-related blood or tissue, which could affect interpretation; this reflects standard clinical practice, and specimens were interpreted by experienced gynecologic pathologists. Follow-up HPV testing and cytology were performed at the first post-re-LEEP visit. HPV clearance was defined as a negative test at this visit and persistence as a positive test; because sequential genotype-level comparison was not performed, true persistence of the original genotype could not be distinguished from reinfection with a new genotype.

### Statistical analysis

Continuous variables were expressed as median (interquartile range [IQR]) or mean +/- SD and compared using the Kruskal-Wallis test; categorical variables were compared using the chi-square or Fisher-Freeman-Halton exact test, with linear-by-linear association tests for ordered categories. Agreement between initial and re-LEEP pathology was assessed with Cohen's kappa. Multivariate logistic regression identified independent predictors of residual CIN2+ disease and receipt of a third surgical intervention, with model fit assessed by the Hosmer-Lemeshow test and Nagelkerke R-squared. Two-sided p<0.05 was considered significant. Analyses used SPSS version 26.0 (IBM Corp., Armonk, NY, USA).

## RESULTS

A total of 287 patients met inclusion criteria. The indication for re-LEEP was a positive surgical margin in 202 patients, combined positive margin and ECC findings in 47, an indeterminate margin in 31, isolated positive ECC in 5, and ASC-H cytology in 2. Mean age was 41.1 +/- 8.6 years (range 22–67), and 45/287 (15.7%) were postmenopausal. Median follow-up after re-LEEP was 3 months (range 1–10, IQR 2–4). No significant differences were observed between margin categories for age, parity, smoking, comorbidities, baseline HPV genotype, or cytology, and specimen size was also similar across groups (median 2.50–2.60 cm, p=0.682) (Table 1).

**Table 1:** Demographic and clinical characteristics, and specimen size, according to initial surgical margin status.

Variable		Margin adjacency (n=5)	Margin continuity (n=246)	Margin not evaluable (n=36)	p-value
Age (years), median (IQR)		45 (36.5–47.5)	41 (35.8–46.0)	41 (34.0–47.8)	0.753
Parity, median (IQR)		2 (1–2)	1 (1–2)	1 (1–2.8)	0.965
LEEP specimen size (cm), median (IQR)		2.50 (1.85–2.90)	2.60 (2.30–3.00)	2.50 (2.23–3.08)	0.682
Smoking, n (%)					
	No	1 (20.0)	93 (37.8)	14 (38.9)	0.070
	Yes	4 (80.0)	153 (62.2)	22 (61.1)	
Comorbidity, n (%)					
	None	4 (80.0)	217 (88.2)	30 (83.3)	0.708
	Hypertension	1 (20.0)	20 (8.1)	2 (5.6)	
	Diabetes mellitus	0	7 (2.8)	4 (11.1)	
	Asthma	0	1 (0.4)	0	
	Breast cancer	0	1 (0.4)	0	
Baseline HPV status, n (%)					
	HPV negative	0 (0.0)	6 (2.4)	1 (2.8)	0.925*
	HPV16/18 (single or multiple)	3 (60.0)	168 (68.3)	26 (72.2)	
	Other high-risk HPV	2 (40.0)	72 (29.3)	9 (25.0)	
Baseline cytology, n (%)					
	Benign	2 (40.0)	70 (28.5)	11 (30.6)	0.780*
	ASC-US	1 (20.0)	73 (29.7)	8 (22.2)	
	LSIL	2 (40.0)	47 (19.1)	4 (11.1)	
	ASC-H	0	25 (10.2)	7 (19.4)	
	HSIL	0	28 (11.4)	5 (13.9)	
	AGC	0	2 (0.8)	0	
	No smear performed	0	1 (0.4)	1 (2.8)	

* Fisher-Freeman-Halton exact test. IQR: interquartile range; LEEP: loop electrosurgical excision procedure; HPV: human papillomavirus; ASC-H: atypical squamous cells, cannot exclude a high-grade squamous intraepithelial lesion.

Initial and re-LEEP pathology showed a significant group-level association (chi-square=17.669, p=0.039; Fisher-Freeman-Halton p=0.004) and a significant linear trend (chi-square=7.282, p=0.007), but Cohen's kappa showed no agreement beyond chance (kappa=-0.009, p=0.754); most patients with initial CIN2/3 had CIN1 at re-LEEP. Margin status was also associated with re-LEEP pathology (Fisher-Freeman-Halton p=0.048; linear-by-linear chi-square=4.324, p=0.038): CIN3 was most common with margin adjacency (60.0%), while CIN1 predominated with margin continuity (50.0%) and non-evaluable margins (47.2%). Glandular involvement was not significantly associated with ECC findings, margin status, or re-LEEP pathology (p=0.218, 0.136, 0.095, respectively), although CIN3 at re-LEEP was numerically more frequent with glandular involvement (19.4 vs. 7.5%) (Table 2).

**Table 2:** Pathology and margin associations at re-loop electrosurgical excision procedure.

A. Initial loop electrosurgical excision procedure pathology vs. re-loop electrosurgical excision procedure pathology					
Initial LEEP pathology	Benign n (%)	CIN1 n (%)	CIN2 n (%)	CIN3 n (%)	Total
Benign (n=3)	1 (33.3)	2 (66.7)	0 (0)	0 (0)	3
CIN1 (n=5)	0 (0)	3 (60.0)	1 (20.0)	1 (20.0)	5
CIN2 (n=83)	17 (20.5)	43 (51.8)	20 (24.1)	3 (3.6)	83
CIN3 (n=196)	32 (16.3)	92 (46.9)	30 (15.3)	42 (21.4)	196
Total (n=287)	50 (17.4)	140 (48.8)	51 (17.8)	46 (16.0)	287


B. Initial surgical margin status vs. re-loop electrosurgical excision procedure pathology					
Surgical margin status	Benign n (%)	CIN1 n (%)	CIN2 n (%)	CIN3 n (%)	Total
Margin adjacency	1 (20.0)	0 (0.0)	1 (20.0)	3 (60.0)	5
Margin continuity	39 (15.9)	123 (50.0)	46 (18.7)	38 (15.4)	246
Margin not evaluable	10 (27.8)	17 (47.2)	4 (11.1)	5 (13.9)	36
Total	50 (17.4)	140 (48.8)	51 (17.8)	46 (16.0)	287


C. Glandular involvement and associations with endocervical curettage, margin status, and re-loop electrosurgical excision procedure pathology			
Variable	Gland involvement (+) n (%)	Gland involvement (-) n (%)	p-value
ECC benign	159 (77.2)	65 (81.3)	
ECC CIN1	4 (1.9)	4 (5.0)	
ECC CIN2	15 (7.3)	2 (2.5)	
ECC CIN3	28 (13.6)	9 (11.3)	0.218*
Margin adjacency	3 (1.5)	2 (2.5)	
Margin continuity	182 (88.3)	64 (80.0)	
Margin not evaluable	21 (10.2)	14 (17.5)	0.136*
Re-LEEP benign	33 (16.0)	16 (20.0)	
Re-LEEP CIN1	95 (46.1)	45 (56.3)	
Re-LEEP CIN2	38 (18.4)	13 (16.3)	
Re-LEEP CIN3	40 (19.4)	6 (7.5)	0.095*


At the first post-re-LEEP visit, performed after a median follow-up of 3 months (IQR 2–4), HPV test negativity—defined as HPV clearance in this study—was observed in 179 of 203 patients (88.2%) with negative re-LEEP margins. Test negativity was highest for HPV16 (91.3%) and lowest for HPV18 (81.8%) and multiple infections (82.1%), without a significant relationship to baseline genotype (p=0.099). In this same cohort, residual CIN2+ disease at repeat excision occurred in 18/63 (28.6%) with initial CIN2 and 37/135 (27.4%) with initial CIN3, an overall rate of 27.1% (55/203).

In multivariate analysis, margin adjacency (OR 11.045, 95%CI 1.015–120.181, p=0.049) and glandular involvement (OR 1.938, 95%CI 1.048–3.584, p=0.035) independently predicted residual CIN2+ disease at repeat LEEP (model p=0.043; Hosmer-Lemeshow p=0.864); non-evaluable margins, specimen size, and initial CIN2+ status were not significant. For receipt of a third intervention, positive margin (OR 333.33, 95%CI 55.56–1000.00, p<0.001) and positive ECC (OR 41.67, 95%CI 7.19–250.00, p<0.001) at repeat LEEP were the strongest independent predictors (model p<0.001; Nagelkerke R-squared=0.77; Hosmer-Lemeshow p=0.427); glandular involvement and residual disease were not significant (Table 3 and Figure 1). Because these predictors arose at different procedural stages and reflected different endpoints, they were integrated only into a preliminary, hypothesis-generating framework rather than a validated clinical risk model (Table 4).

**Table 3:** Multivariate logistic regression analyses of predictors of residual CIN2+ disease at repeat loop electrosurgical excision procedureand of third surgical intervention.

A. Predictors of residual CIN2+ disease at repeat loop electrosurgical excision procedure (initial loop electrosurgical excision procedure factors)				
Variable	Category (reference)	OR [Exp(B)]	95%CI	p-value
Margin adjacency	Vs. continuity	11.045	1.015–120.181	**0.049**
Margin not evaluable	Vs. continuity	1.255	0.550–2.863	0.590
Gland involvement	Present vs. absent	1.938	1.048–3.584	**0.035**
LEEP specimen size (cm)	Continuous	1.176	0.760–1.819	0.467
Initial LEEP CIN2+	Yes vs. no	0.944	0.165–5.410	0.948


B. Predictors of third surgical intervention (findings at repeat/second loop electrosurgical excision procedure)				
Variable	Category (reference)	OR [Exp(B)]	95%CI	p-value
Surgical margin status (re-LEEP)	Positive vs. negative	333.33	55.56–1,000.00	**<0.001**
ECC findings (re-LEEP)	Positive vs. negative	41.67	7.19–250.00	**<0.001**
Gland involvement (re-LEEP)	Present vs. absent	1.838	0.345–9.804	0.476
Residual disease (re-LEEP, CIN2+)	Present vs. absent	1.880	0.368–9.615	0.447


Chi-square (9)=17.669, p=0.039; Fisher-Freeman-Halton, p=0.004; linear-by-linear association, chi-square=7.282, p=0.007; Cohen's kappa=-0.009, p=0.754. LEEP: loop electrosurgical excision procedure.

Fisher-Freeman-Halton, p=0.048; linear-by-linear association, chi-square=4.324, p=0.038.

* Fisher-Freeman-Halton exact test. One patient with missing glandular involvement status was excluded from this analysis (n=286). LEEP: loop electrosurgical excision procedure; ECC: endocervical curettage.

Model p=0.043; Hosmer-Lemeshow p=0.864. Reference categories: margin continuity, absence of glandular involvement. ECC findings were not independently associated (overall p=0.243) and were excluded from the final model. OR: odds ratio; LEEP: loop electrosurgical excision procedure. Bold values indicate statistical significance (p<0.05).

Omnibus test p<0.001; Nagelkerke R2=0.77; Hosmer-Lemeshow p=0.427. Reference categories: negative surgical margin, negative ECC, absence of glandular involvement, absence of residual disease. OR: odds ratio; LEEP: loop electrosurgical excision procedure; ECC: endocervical curettage. Bold values indicate statistical significance (p<0.05).

**Figure 1:**
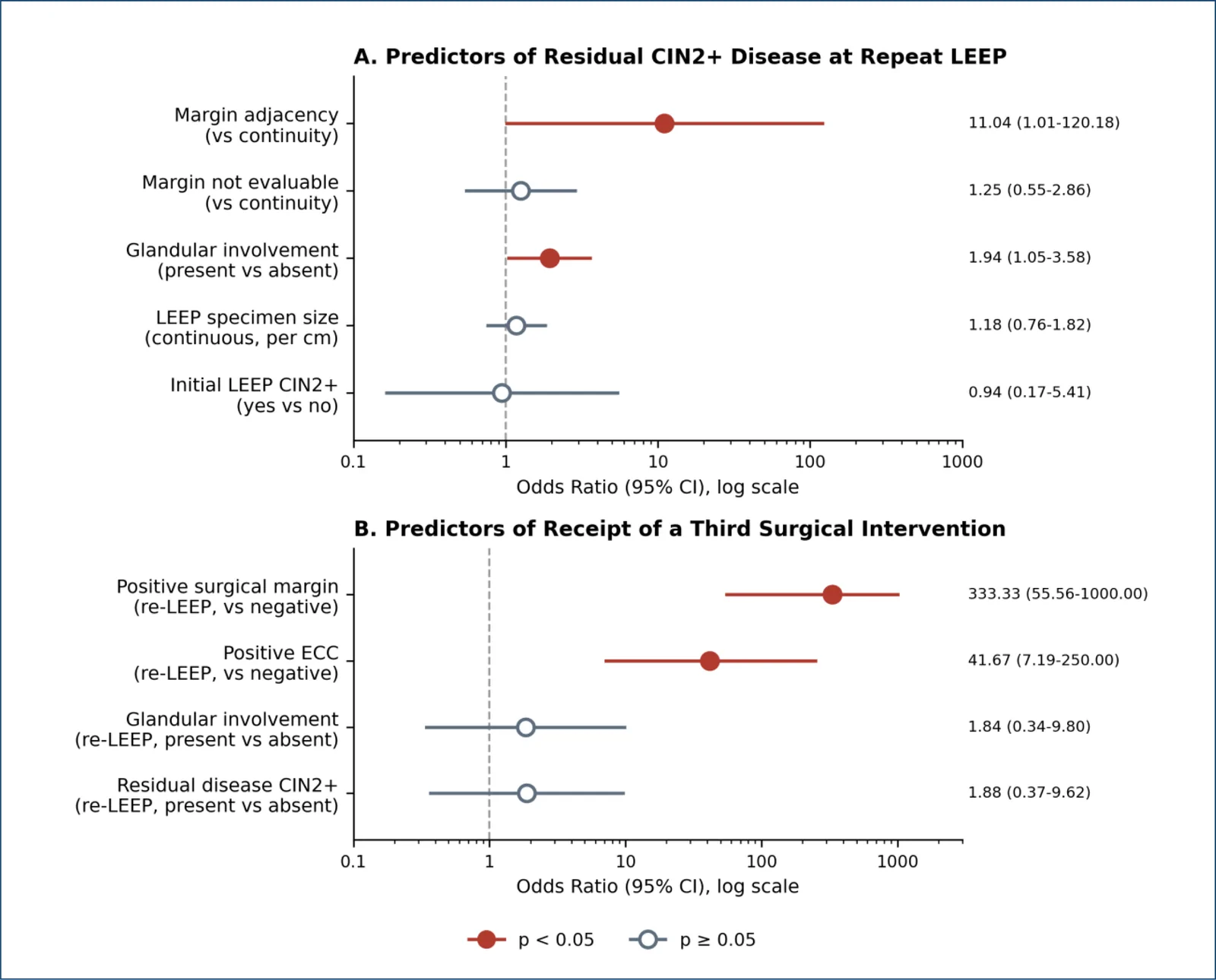
Forest plot of multivariate predictors of (A) residual CIN2+ disease at repeat LEEP and (B) receipt of a third surgical intervention. Points show adjusted odds ratios; horizontal lines show 95% confidence intervals; the dashed vertical line marks OR 1. Filled red markers indicate p<0.05; open gray markers indicate p≥0.05. Panel (A) reflects the model in Table 3A; panel (B) reflects the model in Table 3B.

**Table 4:** Preliminary, hypothesis-generating framework integrating findings across initial loop electrosurgical excision procedure and re-loop electrosurgical excision procedure.

Provisional category	Stage-specific findings	Suggested approach
Lower-risk profile	Initial LEEP: margin continuity and no glandular involvement; re-LEEP: negative margin and negative ECC	Standard cytology/HPV co-testing follow-up
Intermediate profile	Initial LEEP: non-evaluable margin or glandular involvement; re-LEEP: no positive margin or positive ECC	Closer-interval follow-up; consider earlier HPV/cytology reassessment
Higher-risk profile	Initial LEEP: margin adjacency; and/or re-LEEP: positive margin or positive ECC	Close surveillance; multidisciplinary discussion regarding further intervention

LEEP: loop electrosurgical excision procedure; ECC: endocervical curettage; HPV: human papillomavirus.

## DISCUSSION

This study evaluated initial LEEP margin subtype, glandular involvement, and ECC findings as predictors of re-LEEP outcomes and residual disease. Initial and re-LEEP pathology showed a significant group-level association but no case-by-case agreement beyond chance (kappa=-0.009), indicating that initial histopathology did not reliably predict re-LEEP histology at the individual-patient level. This finding is consistent with prior evidence that margin status alone has limited sensitivity for residual or recurrent CIN2+ compared with post-treatment HPV testing^7^. However, the observed discordance should not be interpreted as evidence of a distinct disease process, because the predominance of CIN1 at re-LEEP may simply reflect removal of the bulk of the high-grade lesion during the initial excision. Fernández-Montolí et al. similarly found that HR-HPV positivity and margin involvement independently predicted residual or recurrent CIN2-3 despite limited histopathological concordance^11^, and HPV persistence was the only consistent predictor of recurrence across excision techniques in a 5-year follow-up study^14^. These findings support integrating markers beyond simple pathology concordance into clinical decision-making.

Margin adjacency was the category most closely linked to high-grade residual disease and remained an independent predictor after adjustment (OR 11.045, p=0.049), consistent with Fernández-Montolí et al.'s finding that uncertain margins carried the highest recurrence risk (OR 15.84)^11^ and with Abdulaziz et al.'s report that multifocal margin involvement increased residual disease risk^15^. In contrast, Bruno et al. found cone margin status was not an independent predictor in a postmenopausal CIN3 population^16^, a discrepancy likely reflecting differences in age distribution and margin categorization. Specimen size did not differ across margin categories in our cohort, partly contrasting with Ge et al.'s finding that cone depth >0.5 cm was protective against recurrence^17^, and with Kim et al.'s observation that cone depth and margin involvement varied by whether glandular involvement was recognized before conization^9^.

Glandular involvement has previously been linked to more aggressive management without clearly worse outcomes; Kim et al. found that glandular involvement-positive patients underwent additional procedures more often despite similar residual or recurrence rates^9^, and Paternostro et al. questioned the value of routine ECC, which altered management in only 1.7% of cases and had a negative predictive value of 43.8%^18^. In our cohort, glandular involvement was not associated with ECC findings or margin status but independently predicted residual CIN2+ at repeat LEEP (OR 1.938, p=0.035). ECC positivity has also been reported as the second strongest independent predictor of residual disease after HPV persistence in postmenopausal patients^16^. In our dataset, however, initial ECC was not independently associated with residual CIN2+, whereas positive ECC at re-LEEP was strongly associated with receipt of a third surgical intervention; these findings represent different procedural stages and endpoints and should not be interpreted as a single prognostic effect. These findings extend our previous work, in which HPV subtype was not independently associated with the rate of positive surgical margin after cervical conization^19^, and endocervical glandular involvement did not differ significantly by cone specimen depth^20^.

For receipt of a third intervention, positive margin (OR 333.33) and positive ECC (OR 41.67) at re-LEEP were the strongest predictors, in line with Arbyn et al.'s report of an approximately five-fold increase in treatment failure with positive margins^7^ and Reimers et al.'s finding of a strong association between positive margins and recurrence in human immunodeficiency virus-positive women^21^. That margin and ECC status at the second LEEP were more predictive than initial findings suggests a cumulative effect of risk factors across successive interventions. This finding has practical implications: post-re-LEEP surveillance may warrant closer attention than post-initial-LEEP surveillance, since risk appears to compound rather than reset with each subsequent procedure.

Negative re-LEEP margins did not guarantee HPV test negativity at the first follow-up visit, although negativity reached 88.2% overall and was highest for HPV16 (91.3%) and lowest for HPV18 and multiple infections. This is consistent with Lin et al.'s meta-analysis reporting a 22.7% residual HPV rate after LEEP with negative margins and a 3.6-fold increase in CIN recurrence risk with residual HPV^22^, and with Heydari et al.'s finding that negative HR-HPV combined with clear margins yielded a 98.7% negative predictive value, whereas clear margins alone did not^23^. Persistent HPV positivity has also emerged as the strongest independent predictor of residual disease in postmenopausal CIN3 populations^16^, reinforcing that margin status alone is insufficient; the trend toward genotype-specific differences in HPV test negativity observed here (p=0.099) did not reach significance and would require confirmation in larger cohorts before informing surveillance strategy. Adjunctive molecular strategies may help address this gap: combining HPV testing with p16/Ki-67 dual staining, even on self-sampled material, has been shown to accurately identify women at risk for cervical cancer^24^, suggesting a possible complementary approach to margin- and genotype-based surveillance alone.

Taken together, margin adjacency and glandular involvement at initial LEEP independently predicted residual CIN2+ at re-LEEP, whereas positive margin and positive ECC at re-LEEP were strongly associated with receipt of a third surgical intervention. As a preliminary, hypothesis-generating framework rather than a validated clinical guideline, these stage-specific findings may help structure post-re-LEEP surveillance; prospective validation in an independent cohort is required before clinical adoption.

Strengths of this study include the systematic evaluation of pathological and virological data across two procedures, the analysis of margin subtypes as distinct categories, and separate examination of patients receiving a third intervention, offering more detailed risk stratification than studies limited to binary margin classification. A further strength is the explicit separation of margin adjacency from both frank margin continuity and non-evaluable margins, a distinction blurred in much of the prior literature. Limitations include the retrospective, single-center design, non-standardized repeat HPV genotyping, and short follow-up, which was particularly limiting for assessing HPV clearance and recurrence. The margin adjacency subgroup was small, with a wide confidence interval (OR 11.045, 95%CI 1.015–120.181); pooling it with margin continuity abolished the significant association (OR 0.68, 95%CI 0.30–1.52, p=0.34), indicating the two behave differently, so we retained margin adjacency as a distinct category. Receipt of a third intervention was itself informed by re-LEEP margin and ECC findings, introducing incorporation bias that likely contributes to the very high odds ratios observed for these predictors (OR 333.33 and 41.67); together with the small event numbers and high Nagelkerke R-squared (0.77), these results should be read as exploratory rather than confirmatory, and comparisons were not corrected for multiple testing. Prospective, multicenter validation of the margin subtype classification would help confirm its clinical utility.

## CONCLUSION

Margin subtypes at initial LEEP, particularly margin adjacency, carry significant but case-wise limited value in predicting residual high-grade disease at re-LEEP. Glandular involvement at initial LEEP independently predicted residual CIN2+, whereas positive margin and positive ECC at re-LEEP were strongly associated with receipt of a third surgical intervention. Post-LEEP follow-up should therefore adopt a risk-based approach integrating HPV testing and stage-specific histopathological findings rather than relying on margin status alone; the incremental value of genotype-specific surveillance remains to be established.

## Data Availability

The datasets generated and/or analyzed during the current study are available from the corresponding author upon reasonable request.
